# Approaching expert-level accuracy for differentiating ACL tear types on MRI with deep learning

**DOI:** 10.1038/s41598-024-51666-8

**Published:** 2024-01-10

**Authors:** Yang Xue, Shu Yang, Wenjie Sun, Hui Tan, Kaibin Lin, Li Peng, Zheng Wang, Jianglin Zhang

**Affiliations:** 1 School of Computer Science, Hunan First Normal University, Changsha, 410205 China; 2 Department of Orthopaedic, Hunan Provincial People′s Hospital (The First Affiliated Hospital of Hunan Normal University), Changsha, 410002 China; 3 Hunan Provincial Key Laboratory of Information Technology for Basic Education, Changsha, 410205 China; 4 Department of Radiology, Hunan Provincial People′s Hospital (The First Affiliated Hospital of Hunan Normal University), Changsha, 410002 China; 5 Department of Dermatology, Shenzhen People′s Hospital (The Second Clinical Medical College, Jinan University; The First Affiliated Hospital, Southern University of Science and Technology), Shenzhen, 518020 Guangdong China; 6 Candidate Branch of National Clinical Research Center for Skin Diseases, Shenzhen, 518020 Guangdong China; 7 Department of Geriatrics, Shenzhen People′s Hospital, (The Second Clinical Medical College, Jinan University; The First Affiliated Hospital, Southern University of Science and Technology), Shenzhen, 518020 Guangdong China

**Keywords:** Biomarkers, Health care

## Abstract

Treatment for anterior cruciate ligament (ACL) tears depends on the condition of the ligament. We aimed to identify different tear statuses from preoperative MRI using deep learning-based radiomics with sex and age. We reviewed 862 patients with preoperative MRI scans reflecting ACL status from Hunan Provincial People’s Hospital. Based on sagittal proton density-weighted images, a fully automated approach was developed that consisted of a deep learning model for segmenting ACL tissue (ACL-DNet) and a deep learning-based recognizer for ligament status classification (ACL-SNet). The efficacy of the proposed approach was evaluated by using the sensitivity, specificity and area under the receiver operating characteristic curve (AUC) and compared with that of a group of three orthopedists in the holdout test set. The ACL-DNet model yielded a Dice coefficient of 98% ± 6% on the MRI datasets. Our proposed classification model yielded a sensitivity of 97% and a specificity of 97%. In comparison, the sensitivity of alternative models ranged from 84 to 90%, while the specificity was between 86 and 92%. The AUC of the ACL-SNet model was 99%, demonstrating high overall diagnostic accuracy. The diagnostic performance of the clinical experts as reflected in the AUC was 96%, 92% and 88%, respectively. The fully automated model shows potential as a highly reliable and reproducible tool that allows orthopedists to noninvasively identify the ACL status and may aid in optimizing different techniques, such as ACL remnant preservation, for ACL reconstruction.

## Introduction

The status of the anterior cruciate ligament (ACL)^1^ yields important preoperative information^2^ and is a key metric for prognostic assessment. Orthopedists and radiologists characterize ACL tears into different patterns based on their location along the ligament on MRI^3–5^. The modified Sherman classification utilizes the length of the distal remnant to categorize ACL tears into one of five groups: proximal avulsion tears (distal remnant length > 90% of the total ligament length, Type 1), proximal tears (75% 90%, Type 2), mid-substance tears (25% 75%, Type 3), distal tears (10% 25%, Type 4) or distal avulsion tears (< 10%, Type 5)^6,7^. The accurate characterization of the integrity of the ACL by MRI is important for surgical decision-making and is generally accepted by most osteopathic physicians. Evaluating the ligament remnant length is particularly essential for deciding whether primary ACL repair or advanced ACL reconstruction (ACLR) techniques, such as remnant preservation, augmented remnant repair, repair with bioactive composite scaffolds, biological internal bracing and remnant tensioning, are appropriate for patients before surgery^8–12^. Thus, the MRI classification of ACL tears has great clinical significance for surgeons in terms of technique optimization for different tear types before surgery.

Deep learning (DL) is a promising method for utilizing image information by learning relevant features directly from image signal intensities. DL has been shown to be a valuable method for diagnosing ACL tears^13–16^, particularly in its ability to maximize diagnostic performance while reducing time consumption, subjectivity and errors due to overload and fatigue. Most prior work has focused on classifying injuries^13,14^, detecting abnormalities^17,18^ and automatically segmenting ligaments with a convolutional neural network (CNN)^19^. Radiomics employs advanced computational approaches to convert medical images into quantitative features^20,21^ and provides new perspectives to aid in the diagnosis, recognition, treatment response and prognosis of diseases^22,23^. Deep learning methodologies predicated on image signal intensity encounter limitations in directly integrating data pertinent to the morphology of anterior cruciate ligament (ACL) tissues and the gray level co-occurrence matrix (GLCM) attributes. These attributes are crucial in determining the status of the ligament, a process routinely employed by senior radiologists in clinical assessments. This gap highlights a need for advanced algorithms that can effectively incorporate both intensity and textural feature analyses for more comprehensive and accurate ACL evaluations. Radiomics^24^ employs advanced computations for medical image analysis that decode noninvasive images into quantitative features of tumor phenotypes.

To our knowledge, although several studies have shown the importance of preoperative MRI classifications, no prior studies have used deep learning and radiomics approaches to recognize ACL tear types from preoperative MR images^2,6,25^. Thus, we proposed a novel method for constructing a deep learning-based radiomics with sex and age to precisely recognize the status of the ACL that integrates the following: (i) a system that recognizes abnormalities using deep learning, (ii) a CNN for automated ligament tissue segmentation and (iii) a deep learning-based classifier for ligament status recognition that incorporates 2D images, 2D tissue shape and GLCM radiomics features, age and sex.

## Materials and methods

### MRI datasets and ethical approval

The approval for study protocol and waiver for informed consent was obtained from Hunan Provincial People’s Hospital Ethics Council. All methods were performed in accordance with the relevant guidelines and regulations. Researchers collected the preoperative knee MR images of all subjects who underwent knee arthroscopy from January 2019 to August 2022. Patients who met the following criteria were excluded: age less than 18 years; tumors, chronic ACL tears, partial ACL tears, multiple knee ligament tears or bone fractures around the knee; and diseases that could affect the quality of the ACL, such as metabolic arthritis and knee pigmented villonodular synovitis. The conditions of all subjects were confirmed by arthroscopic pathology, which was considered the reference standard for diagnosis.

Sagittal proton density-weighted spectral attenuated inversion recovery (PDW-SPAIR) MR images were collected from all participants. All participants were scanned in the supine position with a 3.0 T Ingenia and an 8-channel knee coil (Philips Medical Systems, Eindhoven, Netherlands). Imaging parameters for the 3.0 T sagittal PDW-SPAIR sequence included the following: field-of-view (FOV) = 200 mm × 180 mm, echo time (TE) = shortest (automatically selected by the machine), relaxation time (TR) = 1300 ms, slice thickness = 1.5 mm, gap = 0.0 mm, number of signal acquisitions (NSA) = 2.0, voxels = 0.74 × 0.74, matrix = 272 × 243, and flip angle = 90°.

**Figure 1:**
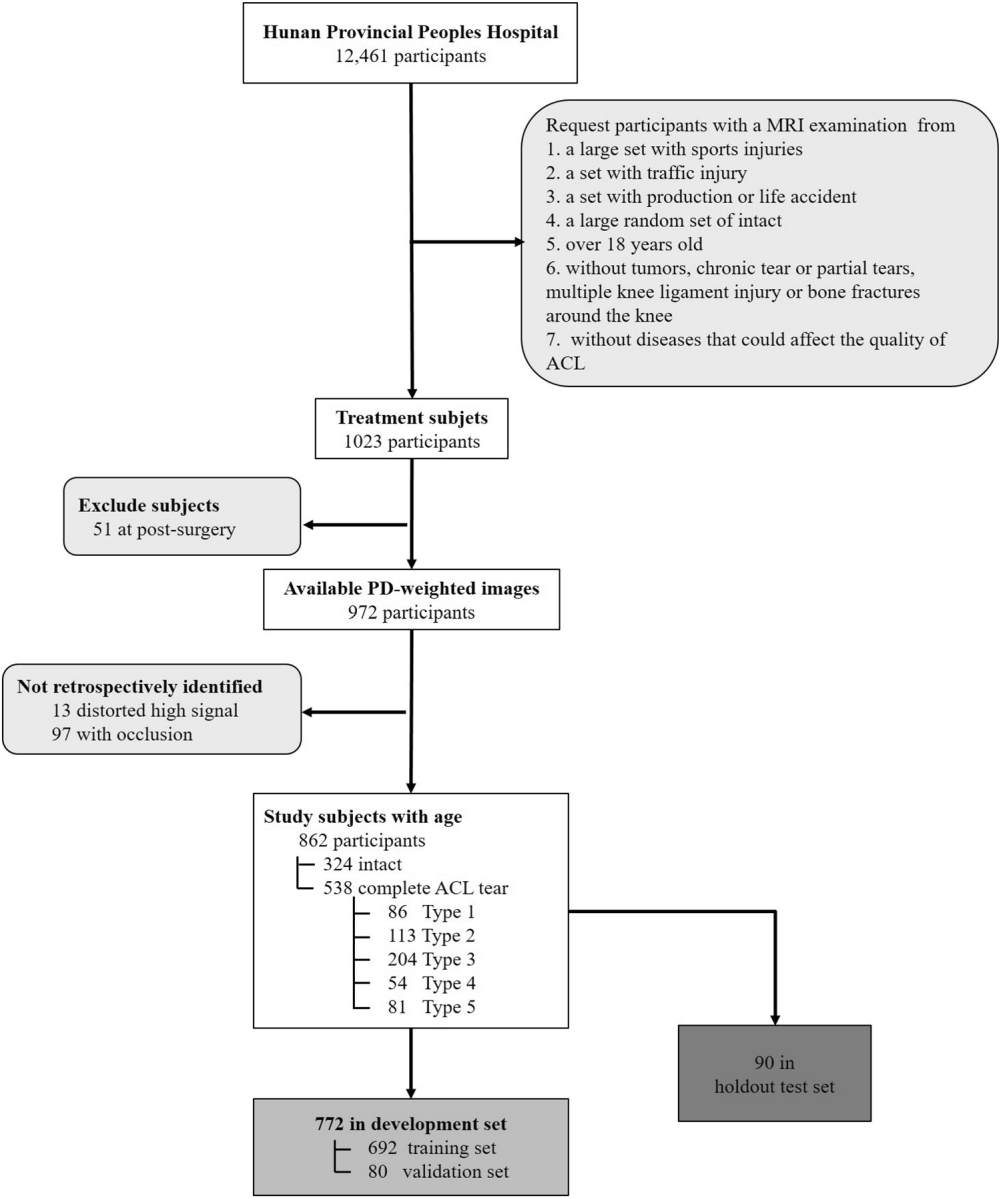
Flowchart illustrating the enrollment criteria. Participant data were used for the development of a deep learning method for the detection of intact (n = 324) and torn (n = 538) ACLs, the latter of which consisted of Type 1 (n = 86), Type 2 (n = 113), Type 3 (n = 204), Type 4 (n = 54) and Type 5 (n = 81) tears. PD = Proton Density.

The participant enrollment process is shown in Fig. 1. Among the 1023 initial participants, 862 participants were finally recruited after we excluded 51 patients whose data were acquired at postsurgery as well as13 patients with distorted high signals and 97 with occlusion of the ACL on the MR images. Among the 862 participants, 324 had intact ACLs, and 538 had ACL tears on their baseline MR images. Table 1 depicts the classification of ligament status for the study cohort. According to ligament status, the participants were randomly allocated into a development set (n = 772) and a holdout test set (n = 90). The development set was further divided into a training set (n = 692) and a validation set (n = 80). Prior to training, the images underwent standardized preprocessing procedures, including normalization and standardization. We implemented data augmentation techniques (such as random clipping, flipping, shifting, tilting, and scaling) was also used to optimize the network’s generalization capability in training phases. During training, regularization methods such as dropout and weight decay were utilized. Throughout the model development and validation phases, early stopping for any signs of bias or overfitting was conducted.

**Table 1:** Ligament status classification.

Status	Description	Location
Healthy	without ACLT	
Complete ACL Tear		
Type 1	Proximal avulsion tear	> 90% distal ligament intact
Type 2	Proximal tear	75–90% distal ligament intact
Type 3	Mid-substance tear	25–75% distal ligament intact
Type 4	Distal tear	10–25% distal ligament intact
Type 5	Distal avulsion tear	< 10% distal ligament intact

### Deep learning for ACL detection

The ACL status recognition system was developed on a Dell XPS 8930 server (hexa-core 3.20 GHz processor, 16 Gb RAM and one NVIDIA GeForce GTX 2080 video card) and implemented in Python (version 2.7, Python Software Foundation, Wilmington, Del). The deep learning models were coded by using the Keras^26^ framework with the TensorFlowGPU 1.15^27^ backend.

**Figure 2:**
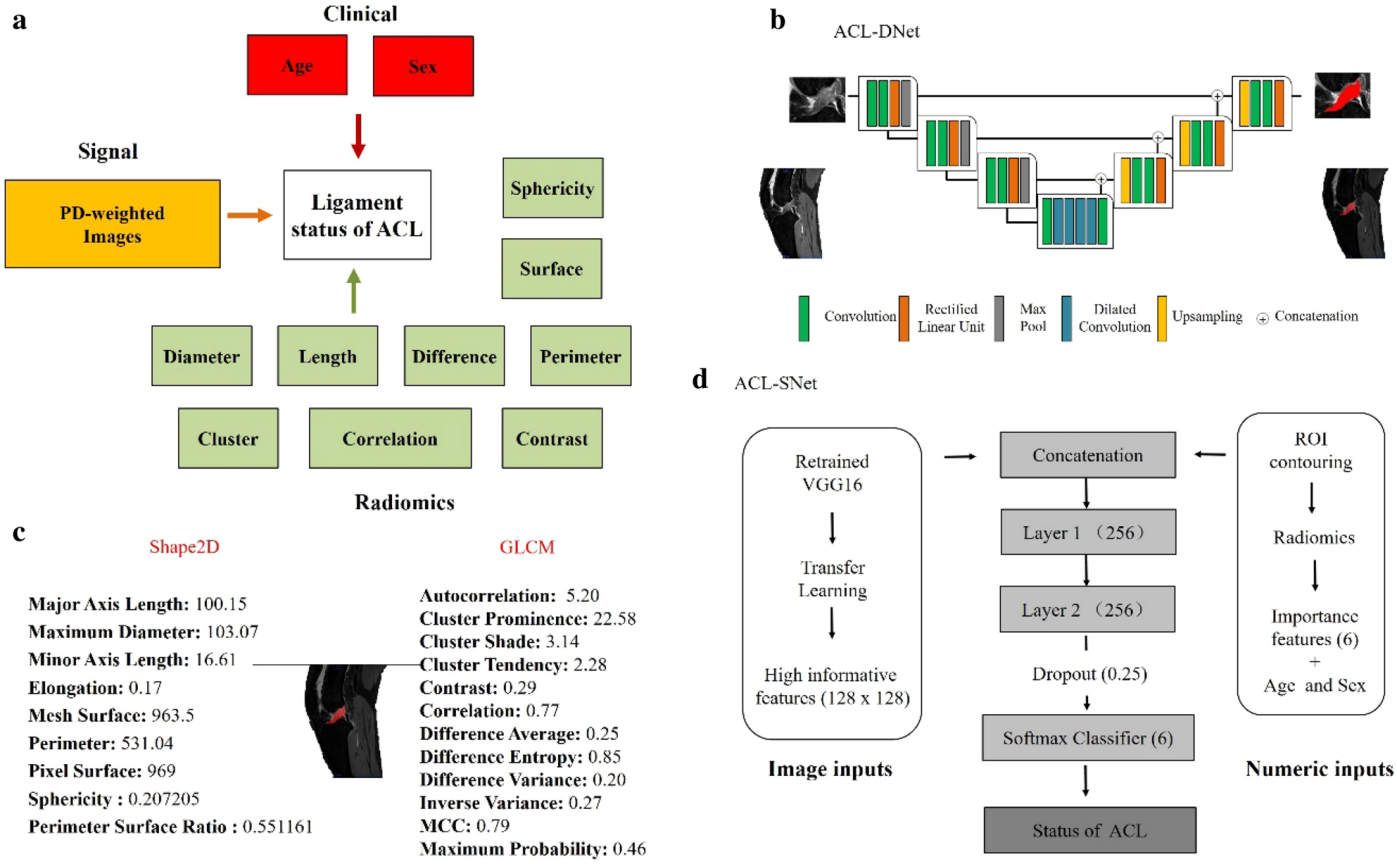
Image demonstrating the overview of the deep learning system. (**a**) Schematic of the deep learning network demonstrates the proposed architecture with a complete set of features used by the deep learning system to differentiate the status of the ACL, which are divided into three categories: clinical, signal and radiomics. (**b**) Schematic of the U-Net architecture used for ACL signal detection (ACL-DNet). (**c**) Multiple quantitative features are calculated for every ligament in every patient, including those shown in this example. These features are stored, providing a rich quantitative description of the tissue. For differential diagnosis, the features are thresholded and then probabilistically fused in a deep learning network. (**d**) Illustration of the hybrid approach for recognizing the ACL status by maximizing the synergy between the image features from the pretrained weights and numeric inputs (ACL-SNet). ACL = Anterior cruciate ligament, GLCM = Gray Level Concurrence Matrix, MCC = Maximal Correlation Coefficient, PD = Proton Density, SPAIR = Spectral Attenuated Inversion Recovery.

The schema consists of three separate components (see Fig. 2a). In our study, the segmentation masks were generated utilizing a Convolutional Neural Network (CNN) based on the U-Net architecture^28,29^, which is particularly adept at medical image segmentation due to its efficiency in learning from a limited amount of data. This network was trained to identify ACL tissue from the MR images, as depicted in Fig. 2b.

The U-Net-based CNN, specifically adapted for this study, takes 512 × 512 × 3 dimension MR images as input and outputs the segmentation of the whole ACL tissue. The segmentation process is automated and leverages edge detection algorithms inherent to the CNN, which have been optimized to recognize the complex anatomy of the knee and the specific texture of ACL tissue in MR images.

The segmentation process performed by the U-Net is not manual but automated; however, it is supervised in the sense that the network was initially trained on a dataset of MR images where the ACL tissue had been manually delineated. This training enables the network to learn the characteristic patterns of the ACL and apply this knowledge to new, unseen images to produce accurate segmentation masks.

**Figure 3:**
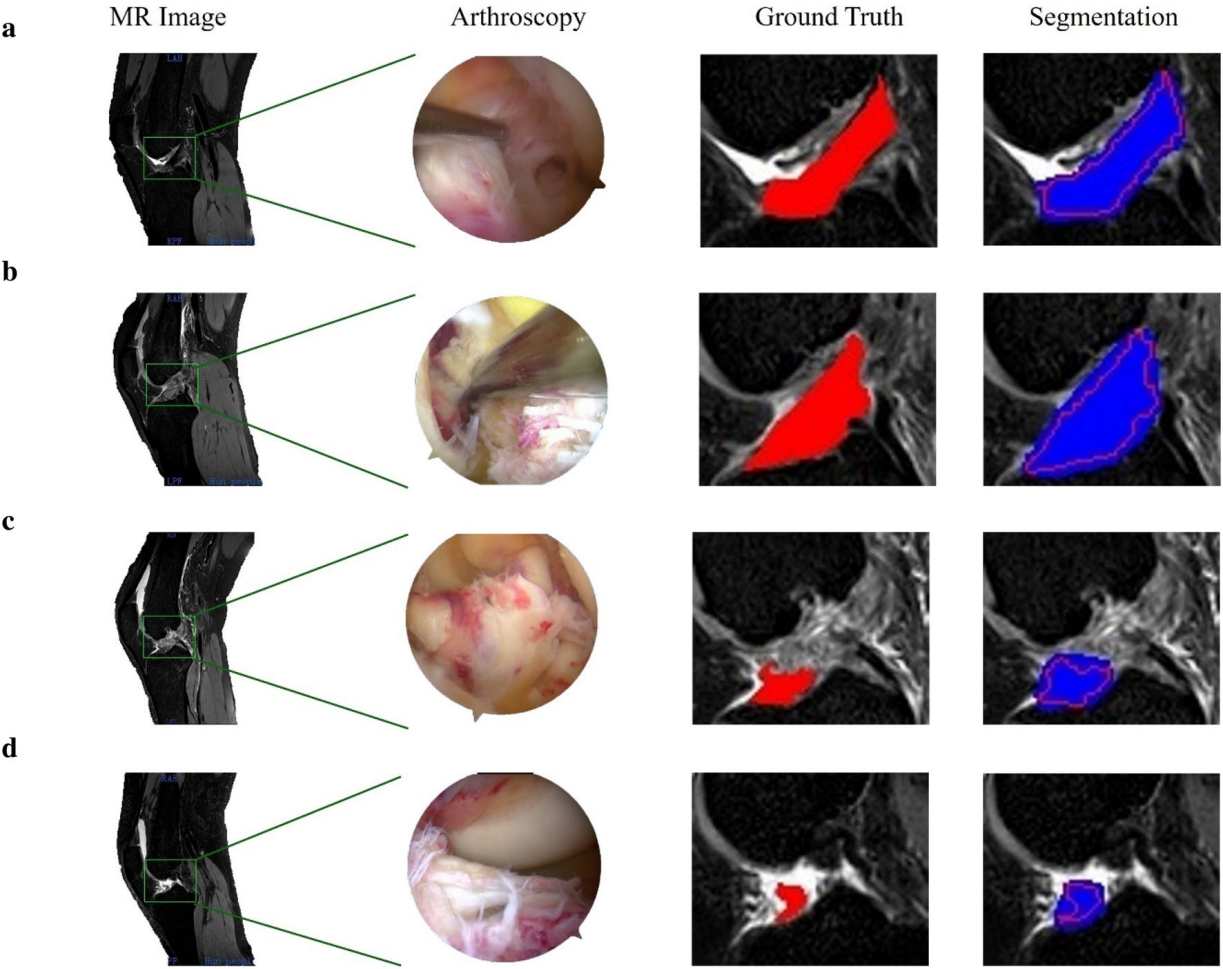
Representative example of ACL tissue segmentation. (**a**) The original grayscale images and segmentation of the ACL of a 19-year-old male patient, which was confirmed as intact with an arthroscopic hook probe. (**b**) The original grayscale images and segmentation for an 18-year-old male patient with a confirmed type 2 ACL tear. (**c**) The original grayscale images and segmentation for a 46-year-old female patient with a confirmed type 4 ACL tear. (**d**) The original grayscale images and segmentation for a 37-year-old female patient with a confirmed type 5 ACL tear. Red indicates the ground truth; blue indicates the prediction by ACL-DNet.

Representative examples of the segmentation results by our deep learning model, referred to as ACL-DNet, are provided in Fig. 3. These examples showcase the model’s ability to accurately delineate the ACL tissue, which is a testament to the robustness of the feature set developed through this process.

### ACL characterization and selection

Image processing was performed by using an open-source Python package (version 2.1.1; https://pyradiomics.readthedocs.io/en/latest/) to extract MR radiomics features^30^ (see Fig. 2c). The segmented ACL mask was overlaid onto each MRI sequence, and the extracted tissue segmentations and a standard atlas were used to extract 21 features of interest for each study (see Fig. 3). Quantitative imaging features (e.g., 2D shape and GLCM) were extracted and then thresholded to obtain qualitative features (e.g., mesh surface, pixel surface, perimeter, maximum diameter, autocorrelation, joint average and cluster prominence).

**Figure 4:**
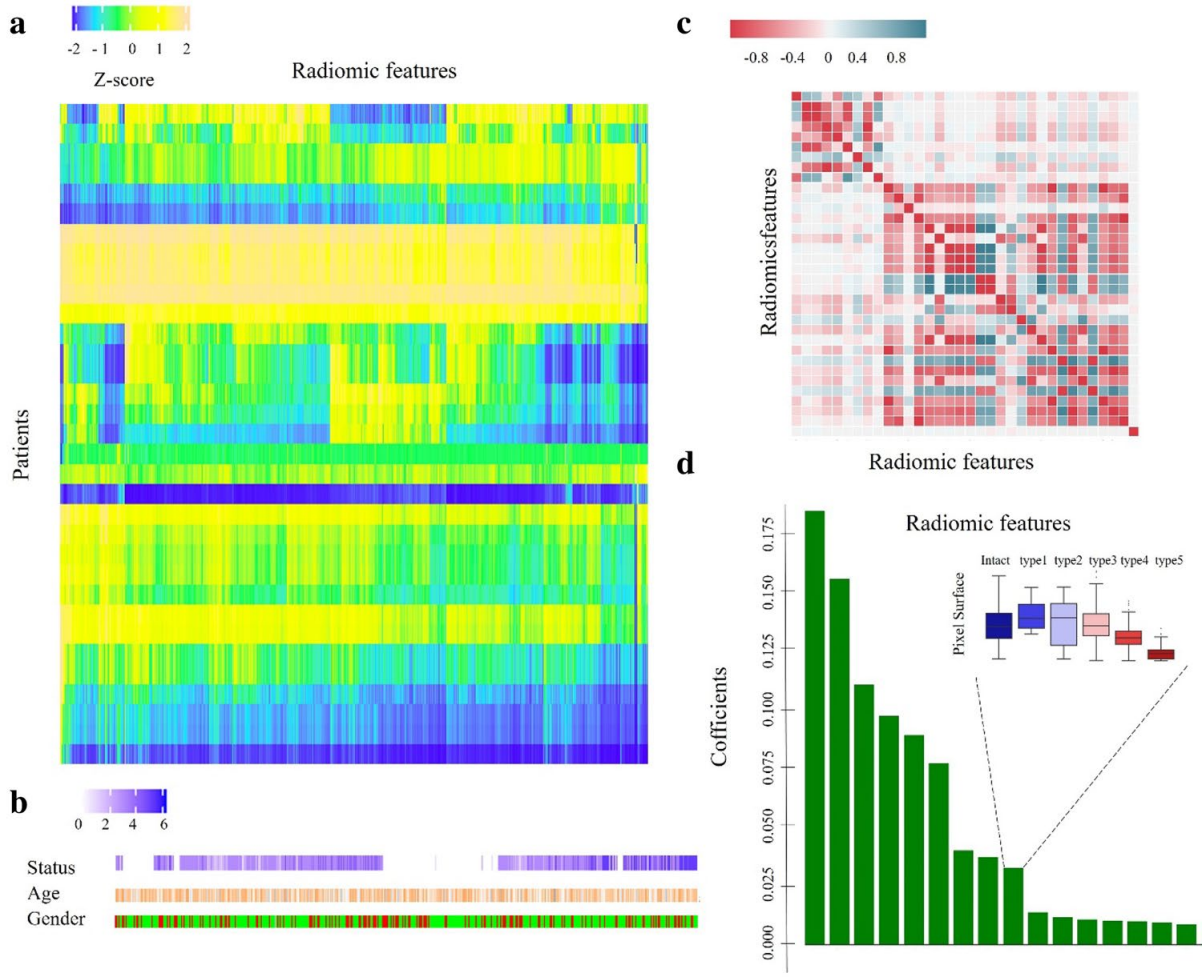
Representations of radiomic features. (**a**) Unsupervised clustering of participants on the x-axis and radiomics feature expression on the y-axis reveals that clustered patients have similar radiomics expression patterns. (**b**) An example showing no correspondence with radiomics expression patterns. (**c**) Correlation coefficient matrix between radiomics variables. (**d**) Ranks and scores identifying important radiomics features.

Unsupervised clustering, Spearman correlation analysis, univariate analysis and feature selection algorithms (FSAs)^31^ were executed for reducing the dimensions of the radiomics features (see Fig. 4). In the Spearman correlation analysis, the thresholds were set to 0.9. Features with *p* < 0.05 for univariate analysis were selected for further analysis. Features were scored based on their ranks provided via a random forest algorithm with tenfold cross-validation strategy, enabling us to reduce their dimensions and select highly discriminative features to identify those important in the differential task^32^. Of these, 6 2D shape features (20%) were selected due to their significant correlation with ACL status (Wilcoxon rank-sum test, *p* < 0.05). The complex heatmap (see Fig. 4a) shows clustering of these features in different clusters of patients.

### Deep learning approaches for developing a differential diagnosis system

For each patient, the image inputs are combined with quantitative inputs (6 radiomics features and 2 clinical factors) by using the pretrained VGG16^33^ as a backbone network for calculating a probability for each diagnosis (ACL-SNet, shown in Fig. 2d). The last fully connected layer of VGG16 at the top of the network is removed, and global max pooling is used to take the maximum values of each layer of the feature maps to transform them into raw values. The pretrained VGG16 first learns the relevant features of the image inputs as a ’warm up’. At the same time, the radiomics features are automatically extracted from the ligament tissue masks.

We used the highly informative pretraining signatures combined with clinical factors and radiomics features as the input of the diagnostic model, which was constructed with 2 dense convolutional layers, 1 dropout (0.25%) layer and a softmax classifier and fine-tuned by the input (a 1D vector of features). The purpose of this study was to decode general preoperative phenotypes present in multiple ACL statuses and encapsulate the differential mapping between features and diseases.

### Performance comparison with radiologists

To compare the performance of the DL system with that of clinical experts, the MRI data were independently and blindly presented to a senior and a junior radiologist with had 5 and 9 years of experience in diagnosis, respectively. The radiologists were given the same MR images and clinical factors available to the DL system and were informed of the equal distribution of diagnoses across patients. One 4th-year orthopedist resident watched the video recording of the knee arthroscopy surgeries and checked the operation records to give the final diagnosis, which was used as the gold standard in this study.

### Evaluation metrics

In our study, we utilized a range of metrics to assess the performance of our deep learning model in the detection and characterization of Anterior Cruciate Ligament (ACL) injuries from MRI images as follows:

*Sensitivity*

1 $${\text{Sensitivity}} = \frac{TP}{{TP + FN}}$$

*Specificity*

2 $${\text{Specificity}} = \frac{TN}{{TN + FP}}$$

*Dice coefficient*

3 $${\text{Dice}} = \frac{2 \times TP}{{FP + 2 \times TP + FN}}$$

*Accuracy*

4 $${\text{Accuracy}} = \frac{TP + TN}{{TP + FP + TN + FN}}$$

The Confusion Matrix was utilized to detail true positives, false positives, true negatives, and false negatives, providing a comprehensive view of classification accuracy. The Area Under the Curve (AUC), with its 95% confidence intervals (CIs) calculated from 2000 iterations, offered an aggregate measure of the model’s performance across all classification thresholds, with probabilities determined by the 'argmax' function. Additionally, a radiomics classifier, developed using a random forest algorithm and tenfold cross-validation on the development set, was employed to evaluate the differential value of quantitative radiomic features. A logistic regression-based regressor was also created for analyzing clinical factors. The differential performance of these components, including a conventional VGG16 model with image inputs, was scrutinized on the test set. Comparisons of the AUCs between the DL-based system and orthopedist evaluations were conducted to ascertain any significant disparities in performance.

## Results

### Participants demographics

A total of 862 patients including 324 (38%) with intact ACLs, 86 with Type 1 ACL tears (10%), 113 with Type 2 tears (13%), 204 Type 3 tears (24%), 54 with Type 4 (6%) and 81 with Type 5 tears (9%) were included in the current study according to the inclusion and exclusion criteria (see Fig. 1). In the entire cohort, there were 483 males (56%) and 379 females (46%), aged 18–78 years (mean age, 36.8 years). The development and test set split resulted in 772 individuals in the development set and 90 in the test set, the latter comprising 34 intact ACLs, 9 Type 1 tears, 12 Type 2 tears; 22 Type 3 statuses, 5 Type 4 tears, and 8 Type 5 tears. No significant difference was found in terms of age (*p* = 0.48) or sex (*p* = 0.39) between the two sets. In terms of ACL status, the intact set showed no difference with the entire ACL tear set (*p* > 0.819). Among the ACL tears, Type 3 tears (31.3%) were significantly more common, and Type 2 tears were the least common (12.8%).

### Performance of DL approach

ACL-DNet segmentation was trained with 30 epochs, producing a Dice coefficient of 0.98 ± 0.06. To compare our system with other previous image segmentation applications^34,35^, we evaluated two state-of-art segmentation DLs, the pyramid scene parsing network (PSPNet^34^)—a commonly used image semantic segmentation network—and a deep convolutional encoder-decoder architecture for image segmentation (SegNet^35^), as demonstrated in Table 2.

**Table 2:** Performance of ACL-DNet vs Alternative DL Systems in ACL Segmentation.

Model	Sensitivity	Specificity	Dice
PSPNet^34^	0.90 (0.80, 0.97)	0.92 (0.85, 0.99)	0.93 (0.90, 0.99)
SegNet^35^	0.84 (0.70, 0.94)	0.86 (0.72, 0.97)	0.88 (0.78, 0.96)
ACL-DNet	0.97 (0.91, 1.00)	0.97 (0.90, 1.00)	0.98 (0.96, 1.00)

The pretrained conventional VGG16 was subjected to 87 epochs of training with image inputs. Among the 21 shape 2D and 6 GLCM radiomics features, showed significant differences according to ACL status, and were used along with age and sex as the quantitative inputs to ACL-SNet. After some of the weights were transfer learned from the pretrained conventional VGG16, ACL-SNet was established following 46 epochs of fine-tuning. Our model achieved an accuracy of 98.8% in the test set. The performances of the conventional VGG16, radiomics classifier, and predictor using sex and age are summarized in Table 3, achieving accuracies of 89.3%, 75.3%, and 68.1%, respectively, in the test set. The automated hybrid model was superior to the individual components in terms of ACL status identification across all datasets.

**Table 3:** Performance of the Conventional VGG16, Radiomics Classifier and Predictor in Identifying ACL Status.

Method	Accuracy (%)		AUC (95% CI)	
Conventional VGG16	89.3	91	84	96
Radiomics classifier	75.3	77	70	83
Predictor (sex and age)	68.1	74	63	85
ACL-SNet	98.8	99	95	99

**Figure 5:**
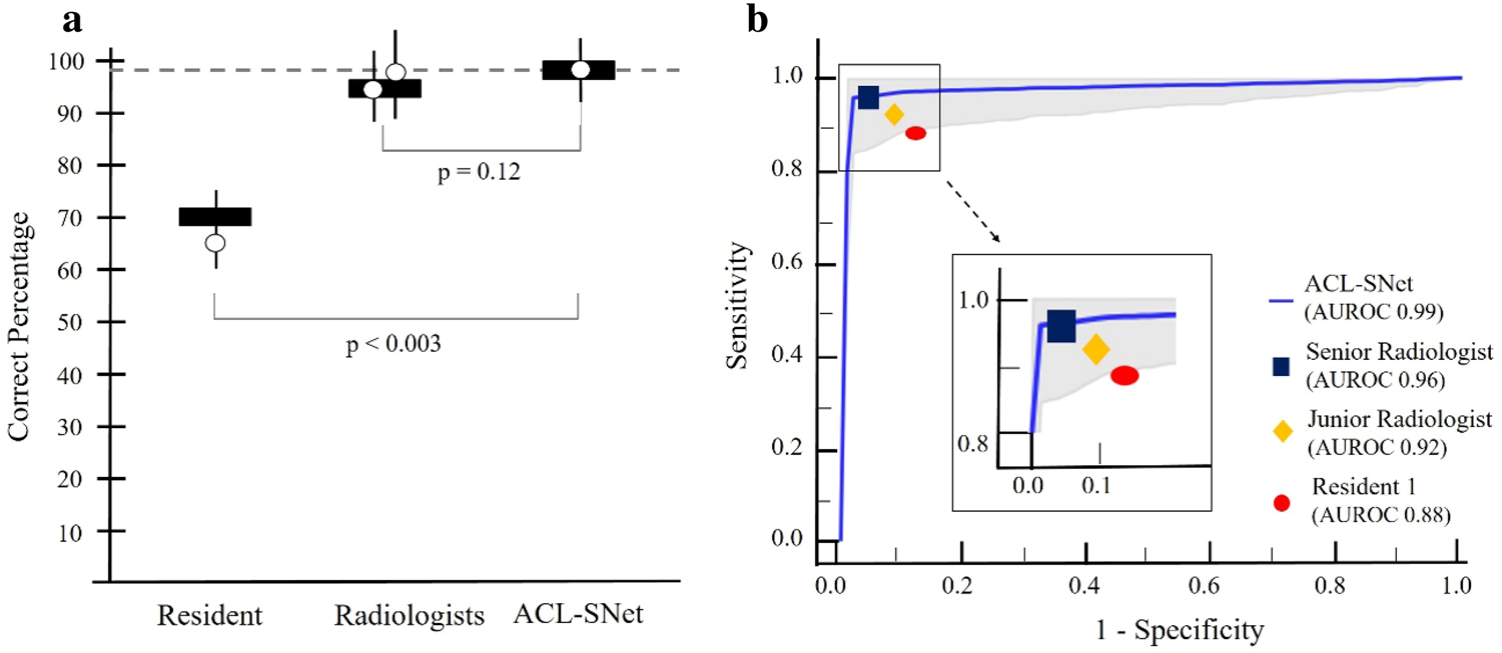
Graphs showing the performance of the hybrid deep learning system and that of the clinical experts. (**a**) Performance is estimated as the percentage correct by listing the differential diagnosis across 90 test patients (6 statuses). Each circle represents a group, and the horizontal line represents the mean across each group. The horizontal dashed line is the performance of the ACL-SNet system. Error bars represent 95% binomial probability confidence intervals. (**b**) Receiver operating characteristic (ROC) curves for the ACL-SNet system (blue) and the clinical experts (other colors). The ACL-SNet system has a similar area under the ROC curve (AUC) to that of radiologists (black and yellow).

The ACL-SNet system performed differential diagnosis for 88 of 90 (97%) patients in the test set. There was no difference in diagnostic accuracy between the ACL-SNet system and senior radiologist on the same set of patients (mean percentage correct across participants, 94%; [95% CI 0.08, 0.32]; *p* = 0.12). The ACL-SNet system performance was better than that of the junior radiologist and orthopedist resident (67–75 of 90 items correct [74–83%]; mean percentage correct across participants, 78.5%; [95% CI 0.06, 0.25]; *p* < 0.003). Comparisons of the ACL-SNet system to the senior radiologist demonstrated similar findings: the AI system performance was similar to that of the clinical expert on all measures and was consistently better than that of the junior radiologist and orthopedist resident in the differential diagnoses (see Fig. 5a).

Receiver operating characteristic curve analysis also revealed that the performance of the ACL-SNet system (AUC, 0.99 [95% CI 0.95, 0.99]) was similar to that of the senior radiologist (AUC, 0.96 [95% CI 0.93, 0.98]), which were both superior to the performance of the junior radiologist (AUC 0.92 [95% CI 0.91, 0.97]) and the orthopedist (AUC 0.88 [95% CI 0.83, 0.907]) (see Fig. 5b).

### Evaluating the clinical experts and DL approach

**Figure 6:**
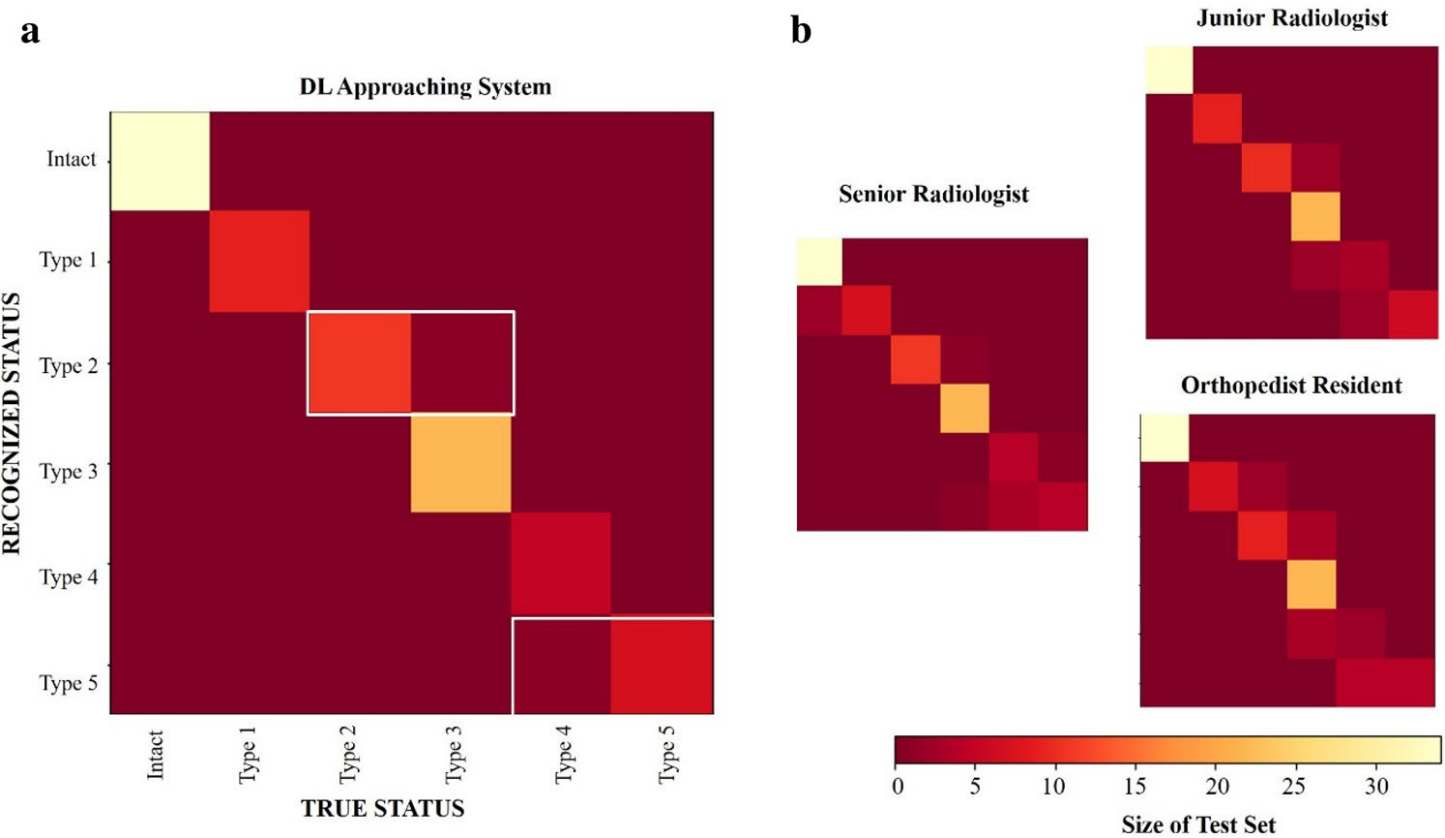
Confusion matrices depict the errors in differential diagnosis by the DL approach system and clinical experts for each disease. In general, the true status is depicted along the x-axis, and the system-/expert-diagnosed status is depicted along the y-axis, with the color bar showing the number of participants whose true status was identified as the corresponding status on the y-axis. A perfect recognition algorithm would result in yellow squares along the diagonal from top left to bottom right. White rectangles on the DL approach system confusion matrix represents mistakes made by the system.

We further evaluated the strengths and weaknesses of the DL system, as well as those of clinical experts, by using confusion matrices. When evaluated in terms of the differential diagnosis with respect to the true status, the DL approach system was found to perform well for most diagnoses but poorly for others (e.g., Type 2 and Type 5 tears, as shown in Fig. 6a). The clinical experts, meanwhile, made errors on a number of different diagnoses (see Fig. 6b). However, the DL approach system and clinical experts made different types of errors. White boxes may indicate a higher frequency of certain types of misclassification, For senior radiologists, junior radiologists, and orthopedic surgery residents, each group showed different misclassification patterns. For example, these patterns may indicate that junior radiologists frequently confuse type 2 and type 3 injuries, whereas orthopedist may have difficulty distinguishing between intact ACL and type 1 injuries. Senior radiologists may have more experience and may show fewer errors overall, but there may still be specific areas where confusion occurs, such as between type 4 and type 5 lesions.

For the majority of cases, the DL system correctly identified the ACL status (88 of 90 patients), meaning that for these correctly diagnosed cases, the DL system had an average of 97% probability of assigning the most likely predicted status. In contrast, in the few cases where the DL system failed to predict the correct ACL status (2 of 90 patients), the system's confidence in the incorrect status was only 3%. This indicates that the system's assessment is significantly less certain about false predictions.

## Discussion

We developed a deep learning approach model to differentiate ACL statuses. Our model incorporates information from 2D ACL image signal intensity, 2D ligament shape features, sex and age into one model in addition to DL-based automated ACL segmentation and fully automated pipeline processes. Although maximum ligament repair is the standard treatment regardless of complete ACL tear status, the preoperative prediction of ACL status is still helpful in guiding treatment and optimizing the appropriate style of operation. Deep learning and radiomics are representative quantitative methodologies for medical image analysis that extract high-dimensional signal features and compute numeric information.

A number studies have applied artificial intelligence and radiomics for diagnosing diseases^36–38^. Rauschecker et al.^36^ developed an AI system that integrated AI-extracted radiomics features into a probabilistic differential diagnosis by using Bayesian inference via data-driven and domain-expertise methodologies. Choi et al.^37^ developed a composite CNN and radiomics approach to predict the isocitrate dehydrogenase (IDH) mutation status of gliomas from preoperative MR images. Here, we constructed a hybrid approach system as a fusion of various methods with complementary strengths that integrates DL-based identification, radiomics features and clinical factors to explore whether the differential accuracy of clinical experts could be improved.

Several studies^13,39–41^ have used deep CNNs for diagnosing ACL injuries. Chang et al.^13^ reported on a multiple CNN for the detection of complete ACL tears. Alexia Tran et al.^39^ build a deep learning-based ACL tear detector. Awan et al.^40^ presented a new deep learning technique for localizing the ACL tear region in MRI images. However, all of these previous studies utilized proton density and T2-weighted sequence MRI scans for the detection of ACL tears. In contrast, the use of conventional MRI is significant as it reflects a more commonly available modality in clinical practice.

Several studies have shown the importance of the preoperative MRI classification of ACL tears, revealing that this classification could affect the choice of surgical technique during the preoperative assessment. MRI classification, for instance, can predict the success rate of the primary ACL repair technique^2,6^; specifically, 90% of type I tears and 88% of type II tears could be repaired, while only 14% of type III tears could be repaired with this technique^2^. For some advanced ACLR techniques, such as remnant preservation, augmented remnant repair, repair with bioactive composite scaffolds, and remnant tensioning, preoperative MRI classification is also crucial because all these techniques involve the remnant ACL and require a sufficient remnant length^8–12,25^. The remnant preservation technique only showed better results in patients with remnant lengths > 20%, which means that patients with MRI classifications of types 1 and 5 tears may not be suitable for this method^42^. Additionally, the biological internal bracing technique can only be utilized for patients classified with a type 1 or type 2 ACL tear, and the relevant MRI assessment must be completed before surgery^43^.

In this study, our proposed approach has been tailored to address certain limitations observed in previous methods, particularly those related to the incorporation of ACL tissue morphology and textural features into the analysis. While image signal intensity provides valuable information, it does not encompass the entirety of diagnostic features a senior radiologist would consider. The DL approach system was investigated as a way to fuse the perceptual and cognitive information from radiologic images. First, a CNN was trained on MR images to detect the ACL. Then, quantitative image and ACL features were explicitly derived by using image processing methodologies. Finally, this information was fused with a small number of clinical factors by using a pretrained classification model to yield the differential diagnosis and classification, which can benefit surgeons’ preoperative decision-making regarding the ACL reconstruction technique.

The combination of DL-based ACL identification, radiomics features and clinical factors enhanced the identification performance in our study, and it consistently yielded better performance than the use of single modalities. The deep learning approach system achieved high diagnostic performance in recognizing the status of the ACL, with an AUC of 0.99. Furthermore, there was no statistically significant difference between the proposed system and clinical experts with different levels of experience in recognizing ACL status.

Our study presents several limitations. Primarily, it employs a cascaded system of two separate deep learning models rather than a unified end-to-end network. While this dual-model structure adds to the training complexity, it offers the advantage of modular adaptability for diverse applications. Additionally, the study relies exclusively on conventional MR images, omitting advanced techniques like perfusion and diffusion-weighted imaging. Though this limits the scope of detectable features, conventional MRI remains more accessible and practical. Furthermore, our dataset does not encompass rare or unique ACL tear types, a constraint that may affect the model’s comprehensive diagnostic capabilities. To address these gaps, future research could explore synthetic data augmentation, transfer learning, and few-shot learning methods to enhance the model's performance with limited data samples. We also propose the development of multicenter collaborations to accumulate a more varied dataset, including rare ACL tear types, thereby broadening the model’s learning spectrum and diagnostic applicability.

In conclusion, we developed a model utilizing deep learning and radiomics techniques that can reliably identify the status of the ACL and classify ACL tears using a fully automated process based on MR imaging. Our model has the potential to be used in the clinic for the noninvasive characterization of ACL tissue to support personalized treatment planning.

## Data Availability

The datasets used and/or analysed during the current study available from the corresponding author on reasonable request.

## References

[1] Daniels SP, van der List JP, Kazam JJ, DiFelice GS (2018). Arthroscopic primary repair of the anterior cruciate ligament: What the radiologist needs to know. Skelet. Radiol..

[2] van der List JP, DiFelice GS (2018). Preoperative magnetic resonance imaging predicts eligibility for arthroscopic primary anterior cruciate ligament repair. Knee Surg. Sports Traumatol. Arthrosc..

[3] Sherman M, Lieber L, Bonamo J, Podesta L, Reiter I (1991). The long-term followup of primary anterior cruciate ligament repair: Defining a rationale for augmentation. Am. J. Sports Med..

[4] Anderson F, Wright M, Anderson M, Alexander F, Popa G, Ahmad C (2020). Inter and intraobserver reliability between orthopaedic surgeons for reparability of the anterior cruciate ligament using mri. Orthop. J. Sports Med..

[5] Virts (1988). Tears of the anterior cruciate ligament and menisci of the knee: Mr imaging evaluation. Radiology.

[6] Mehier C, Ract I, Metten M-A, Najihi N, Guillin R (2022). Primary anterior cruciate ligament repair: Magnetic resonance imaging characterisation of reparable lesions and correlation with arthroscopy. Eur. Radiol..

[7] van der List JP, Mintz DN, DiFelice GS (2017). The location of anterior cruciate ligament tears: A prevalence study using magnetic resonance imaging. Orthop. J. Sports Med..

[8] Hong L, Li X, Zhang H, Liu X, Zhang J, Shen JW, Feng H (2012). Anterior cruciate ligament reconstruction with remnant preservation: A prospective, randomized controlled study. Am. J. Sports Med..

[9] Jung Y-B, Jung H-J, Siti H-T, Lee YS, Lee H-J, Lee SH, Cheon H-Y (2011). Comparison of anterior cruciate ligament reconstruction with preservation only versus remnant tensioning technique. Arthrosc. J. Arthrosc. Relat. Surg..

[10] Leeberg V, Lekdorf J, Wong C, Sonne-Holm S (2014). Tibial eminentia avulsion fracture in children-a systematic review of the current literature. Dan Med. J..

[11] Mackay GM, Blyth MJG, Anthony I, Hopper GP, Ribbans WJ (2015). A review of ligament augmentation with the internalbrace: The surgical principle is described for the lateral ankle ligament and acl repair in particular, and a comprehensive review of other surgical applications and techniques is presented. Surg. Technol. Int..

[12] Murray MM, Flutie BM, Kalish LA, Ecklund K, Fleming BC, Proffen BL, Micheli LJ (2016). The bridge-enhanced anterior cruciate ligament repair (bear) procedure: An early feasibility cohort study. Orthop. J. Sports Med..

[13] Chang PD, Wong TT, Rasiej MJ (2019). Deep learning for detection of complete anterior cruciate ligament tear. J. Digit. Imag..

[14] Awan M, Rahim M, Salim N, Mohammed M, Zapirain B, Abdulkareem K (2021). Efficient detection of knee anterior cruciate ligament from magnetic resonance imaging using deep learning approach. Diagnostics.

[15] Kara AC, Hardalac F (2021). Detection and classification of knee injuries from mr images using the mrnet dataset with progressively operating deep learning methods. Mach. Learn. Knowl. Extract..

[16] Kapoor, V., Tyagi, N., Manocha, B., Arora, A., Roy, S., Nagrath, P. Detection of anterior cruciate ligament tear using deep learning and machine learning techniques. 9–22 (2021).

[17] Bien N, Rajpurkar P, Ball RL, Irvin J, Lungren MP (2018). Deep-learning-assisted diagnosis for knee magnetic resonance imaging: Development and retrospective validation of mrnet. PLoS Med..

[18] Liu F, Guan B, Zhou Z, Samsonov A, Rosas H, Lian K, Sharma R, Kanarek A, Kim J, Guermazi A, Kijowski R (2019). Fully automated diagnosis of anterior cruciate ligament tears on knee mr images by using deep learning. Radiol. Artif. Intell..

[19] Flannery SW, Kiapour AM, Edgar DJ, Murray MM, Beveridge JE, Fleming BC (2021). A transfer learning approach for automatic segmentation of the surgically treated anterior cruciate ligament. J. Orthop. Res..

[20] Kumar V, Gu Y, Basu S, Berglund A, Eschrich SA, Schabath MB, Forster K, Aerts HJWL, Dekker A, Fenstermacher D, Goldgof DB, Hall LO, Lambin P, Balagurunathan Y, Gatenby RA, Gillies RJ (2012). Radiomics: The process and the challenges. Magn. Reson. Imag..

[21] Liu Z, Wang S, Dong D, Wei J, Fang C, Zhou X, Sun K, Li L, Li B, Wang M, Tian J (2019). The applications of radiomics in precision diagnosis and treatment of oncology: Opportunities and challenges. Theranostics.

[22] Mao B, Zhang L, Ning P, Ding F, Ma J (2020). Preoperative prediction for pathological grade of hepatocellular carcinoma via machine learningcbased radiomics. Eur. Radiol..

[23] Ferreira Junior JR, Koenigkam-Santos M, Cipriano F, Fabro AT, Azevedo-Marques PMD (2018). Radiomics-based features for pattern recognition of lung cancer histopathology and metastases. Comput. Methods Progr. Biomed..

[24] Aerts HJ, Velazquez ER, Leijenaar RT, Parmar C, Grossmann P, Carvalho S, Bussink J, Monshouwer R, Haibe-Kains B, Rietveld D (2014). Decoding tumour phenotype by noninvasive imaging using a quantitative radiomics approach. Nat. Commun..

[25] Ahn JH, Lee SH, Choi SH, Lim TK (2010). Magnetic resonance imaging evaluation of anterior cruciate ligament reconstruction using quadrupled hamstring tendon autografts: Comparison of remnant bundle preservation and standard technique. Am. J. Sports Med..

[26] Manaswi, N. K. Understanding and working with keras 10.1007/978-1-4842-35164 (Chapter 2) 31–43 (2018).

[27] Abadi, M., Agarwal, A., Barham, P., Brevdo, E., Chen, Z., Citro, C., Corrado, G. S., Davis, J. D., Devin, M. *et al.* Tensorflow: Large-scale machine learning on heterogeneous distributed systems, arXiv preprint arXiv:1603.04467.

[28] Ronneberger, O., Fischer, P., Brox, T. U-net: Convolutional networks for biomedical image segmentation. In *International Conference on Medical image computing and computer-assisted intervention*.234–241 (2015).

[29] Wang Z, Meng Y, Weng F, Chen Y, Zhang J (2020). An effective cnn method for fully automated segmenting subcutaneous and visceral adipose tissue on CT scans. Ann. Biomed. Eng..

[30] van Griethuysen JJM, Fedorov A, Parmar C, Hosny A, Aucoin N, Narayan V, Beets-Tan RGH, Fillion-Robin J-C, Pieper S, Aerts HJWL (2017). Computational radiomics system to decode the radiographic phenotype. Cancer Res..

[31] Salmanpour MR, Shamsaei M, Rahmim A (2021). Feature selection and machine learning methods for optimal identification and prediction of subtypes in parkinson’s disease. Comput. Methods Progr. Biomed..

[32] Zwanenburg A, Leger S, Agolli L, Pilz K, Troost EG, Richter C, Lock S (2019). Assessing robustness of radiomic features by image perturbation. Sci. Rep..

[33] Simonyan, K., Zisserman, A. Very deep convolutional networks for large-scale image recognition. arXiv preprint arXiv:1409.1556.

[34] Zhao, H., Shi, J., Qi, X., Wang, X., & Jia, J. Pyramid scene parsing network. In *Proceedings of the IEEE Conference on Computer Vision and Pattern Recognition*. 2881–2890 (2017).

[35] Badrinarayanan V, Kendall A, Cipolla R (2017). Segnet: A deep convolutional encoderdecoder architecture for image segmentation. IEEE Trans. Pattern Anal. Mach. Intell..

[36] Rauschecker AM, Rudie JD, Xie L, Wang J, Duong MT, Botzolakis EJ, Kovalovich M, Egan J, Cook TC, Bryan RN (2020). Artificial intelligence system approaching neuroradiologist-level differential diagnosis accuracy at brain mri. Radiology.

[37] Choi YS, Bae S, Chang JH, Kang S-G, Kim SH, Kim J, Rim TH, Choi SH, Jain R, Lee S-K (2021). Fully automated hybrid approach to predict the idh mutation status of gliomas via deep learning and radiomics. Neuro-oncology.

[38] Hu Y, Xie C, Yang H, Ho JW, Wen J, Han L, Lam K-O, Wong IY, Law SY, Chiu KW (2021). Computed tomography-based deep-learning prediction of neoadjuvant chemoradiotherapy treatment response in esophageal squamous cell carcinoma. Radiotherapy Oncol..

[39] Tran A, Lassalle L, Zille P, Guillin R, Pluot E, Adam C, Charachon M, Brat H, Wallaert M, Assignies G (2022). Deep learning to detect anterior cruciate ligament tear on knee mri: Multi-continental external validation. Eur. Radiol..

[40] Awan MJ (2023). MGACA-Net: A novel deep learning based multi-scale guided attention and context aggregation for localization of knee anterior cruciate ligament tears region in MRI images. PeerJ Comput. Sci..

[41] Awan MJ (2021). Improved deep convolutional neural network to classify osteoarthritis from anterior cruciate ligament tear using magnetic resonance imaging. J. Personal. Med..

[42] Lee B-I, Kwon S-W, Kim J-B, Choi H-S, Min K-D (2008). Comparison of clinical results according to amount of preserved remnant in arthroscopic anterior cruciate ligament reconstruction using quadrupled hamstring graft. Arthrosc. J. Arthroscop. Relat. Surg..

[43] Pardiwala DN, Lee D (2023). Biological internal bracing with remnant repair for subacute acl femoral avulsions. J. ISAKOS.

